# Experimental Evidence and Clinical Implications of Pituitary Adenoma Stem Cells

**DOI:** 10.3389/fendo.2020.00054

**Published:** 2020-02-20

**Authors:** Roberto Würth, Stefano Thellung, Alessandro Corsaro, Federica Barbieri, Tullio Florio

**Affiliations:** ^1^Section of Pharmacology, Dipartimento di Medicina Interna and Centro di Eccellenza per la Ricerca Biomedica (CEBR), Università di Genova, Genoa, Italy; ^2^IRCCS Ospedale Policlinico San Martino, Genoa, Italy

**Keywords:** pituitary, cancer stem cells, self-renewal, tumorigenesis, drug sensitivity

## Abstract

Pituitary adenomas, accounting for 15% of diagnosed intracranial neoplasms, are usually benign and pharmacologically and surgically treatable; however, the critical location, mass effects and hormone hypersecretion sustain their significant morbidity. Approximately 35% of pituitary tumors show a less benign course since they are highly proliferative and invasive, poorly resectable, and likely recurring. The latest WHO classification of pituitary tumors includes pituitary transcription factor assessment to determine adenohypophysis cell lineages and accurate designation of adenomas, nevertheless little is known about molecular and cellular pathways which contribute to pituitary tumorigenesis. In malignant tumors the identification of cancer stem cells radically changed the concepts of both tumorigenesis and pharmacological approaches. Cancer stem cells are defined as a subset of undifferentiated transformed cells from which the bulk of cancer cells populating a tumor mass is generated. These cells are able to self-renew, promoting tumor progression and recurrence of malignant tumors, also conferring cytotoxic drug resistance. On the other hand, the existence of stem cells within benign tumors is still debated. The presence of adult stem cells in human and murine pituitaries where they sustain the high plasticity of hormone-producing cells, allowed the hypothesis that putative tumor stem cells might exist in pituitary adenomas, reinforcing the concept that the cancer stem cell model could also be applied to pituitary tumorigenesis. In the last few years, the isolation and phenotypic characterization of putative pituitary adenoma stem-like cells was performed using a wide and heterogeneous variety of experimental models and techniques, although the role of these cells in adenoma initiation and progression is still not completely definite. The assessment of possible pituitary adenoma-initiating cell population would be of extreme relevance to better understand pituitary tumor biology and to identify novel potential diagnostic markers and pharmacological targets. In this review, we summarize the most updated studies focused on the definition of pituitary adenoma stem cell phenotype and functional features, highlighting the biological processes and intracellular pathways potentially involved in driving tumor growth, relapse, and therapy resistance.

## Adult Stem Cells VS. Cancer Stem Cells

Tissue-resident adult stem cells are undifferentiated cells able to self-renew for long periods by asymmetric division, and to generate the bulk of differentiated cells within tissues to support organ repair and regeneration (1). Adult stem cells fate and functioning greatly depend on the stem cell niche, a specialized surrounding microenvironment which control stem cell homing and the balance between self-renewal and differentiation (2). In general, healthy stem cells show a stable diploid genome, slow growth rate or quiescence, granting their persistence for the lifetime of the organism, and express pattern of surface markers distinctive for stem cells in different organs (3). Before having a complete phenotype maturation, stem cells develop into partly differentiated progenitors or committed cells (4).

Tumors are characterized by cell heterogeneity including cell populations with stem-like features, whose identification have revolutionized the concept of cancer origin: according to the hierarchical model of tumorigenesis, tumors arise from a pool of self-renewing cancer stem cells (CSCs) which initiate and maintain tumor growth, and establish a differentiation hierarchy by generating non-tumorigenic differentiated cancer cell bulk. Stem cells, immature progenitors, or more differentiated progenies that develop oncogenic mutations, followed by the accumulation of multiple genetic, epigenetic and microenvironmental hits, acquire a fully transformed phenotype which, associated to typical stem cell features, gives origin to tumor specific CSCs (5). Importantly, hierarchical organization is reversible due to cell plasticity of differentiated cancer cells which can de-differentiate to regenerate CSCs and sustain tumor progression (6). CSCs have been identified in both hematopoietic and solid tumors, showing unlimited self-renewal and proliferative potential also in the absence of extracellular cues, pluripotency, expression of stemness markers, and aneuploidy. CSCs, as well as normal stem cells, have improved DNA-repair and detoxification mechanisms, and high activity of anti-apoptotic and pro-survival pathways. Thus, CSCs are able to resist to anticancer drugs determining chemoresistance, and supporting tumor progression and relapse (7). Migratory capacity is also a common feature of normal stem cells and it is functional to cell homing and tissue regeneration (8), whereas the high motility of CSCs favors their invasive and metastatic capacity. Differently from normal stem cells, during tumor progression CSCs build their own altered niche, recruiting neighboring stromal, mesenchymal, endothelial, and immune cells; this delicate microenvironment further protects and favors CSC maintenance and proliferation (9). Although normal stem cells and CSCs share key signaling pathways and transcription factors which regulate self-renewal and determine cell fate (Wnt/β-catenin, BMPs, Sonic hedgehog (SHH), Notch, Sox2, Oct4, Nanog, Bmi-1, PI3K/Akt cascade), in normal stem cells this signaling tightly depends on extrinsic growth factor signals, while in CSCs, dysregulation or hyperactivation of transcription factor-mediated pathways drives abnormal self-renewal and tumorigenesis, sustaining differentiation into highly proliferative cancer cells (10).

Normal stem cells can be experimentally identified and isolated according to the expression of specific stem cell antigens (e.g., SOX2, NANOG, OCT4, CD44) and the *in vitro* clonogenic and differentiation potentials; however, CSC characterization, beside these parameters, requires the establishment of the tumorigenic potential in *in vivo* models.

CSC-dependent intratumor cellular heterogeneity has been typically considered the basis for malignant tumor development, progression and spreading. However, in recent years, several studies reported the characterization of CSC-like subpopulation also in benign tumors [i.e., meningiomas (11) and pituitary adenomas (12)], highlighting the possibility that, similarly to normal organogenesis during development, tumorigenesis of all kinds of neoplasia, either benign or malignant, requires the activity of stem-like cell subpopulations (13).

## An Overview of the Human Pituitary Gland

Pituitary is the main endocrine regulatory gland, which transmit hypothalamic signals to target organs through systemic hormone secretion. It is composed of anterior (adenohypophysis) and posterior (neurohypophysis) lobes with distinct embryological origin, morphology and functions; the endocrine center is adenohypophysis whose specialized cell types are identified according to the secreted hormone: corticotroph cells produce adrenocorticotropic hormone (ACTH); gonadotrophs, follicle-stimulating (FSH) and luteinizing (LH) hormones; lactotrophs, prolactin (PRL); somatotrophs, growth hormone (GH), and thyrotrophs, thyroid-stimulating hormone (TSH). Undifferentiated pituitary stem cells (PSCs) exist within anterior pituitary and give rise to the three main progenitor lineages, characterized by the expression of essential transcription factors for lineage commitment and terminal differentiation: (i) PIT1-positive PSCs differentiate into GH/PRL/TSH-expressing cells, (ii) TPIT-cells originate the ACTH-secreting subpopulation, while (iii) LH/FSH-secreting cells derive from SF1 lineage (14). Secretory cell types also include somato-lactotrophs which release both GH and PRL, and are considered less differentiated precursors of the mature cells releasing the specific hormone. Neurohypophysis receives peptide hormones (anti-diuretic hormone and oxytocin) via axonal terminals of neurons projecting from the hypothalamus, and releases them under the hypothalamic control.

Adenohypophysis also contains endothelial cells and pericytes, and non-endocrine S100β-positive folliculo-stellate (FS) cells which produce growth factors and cytokines regulating and sustaining hormonal cell activity by integrating paracrine signals (15). FS cells maintain pituitary homeostasis, favoring the maturation of stem/progenitor cells and have been considered as putative PSCs (16, 17). Indeed, the fine tuning of number and activity of secretory cell types in different physiologic conditions requires high plasticity of the pituitary gland and is based on the presence of stem and progenitor cells, which control cell turnover and differentiation (18).

### Pituitary Stem Cells in Cell Turnover and Responses to Hormones

Adult pituitary gland plasticity grants continuous cell turnover (homeostasis), and dynamically adapts its activity to either physiological cues (during puberty somatotroph cell number increase, or during pregnancy and lactation an increase in lactotrophs is observed) or pathological damages, by increasing specific hormone production. This process involves the recruitment of non-hormonal pituitary stem/progenitor cells (16), which are characterized by the expression of stem cell markers (Oct4, Nanog), the growth as spheroids (“pituispheres”), and the ability to differentiate into pituitary secretory cells. For example, the activity of these subpopulations gives rise to the generation of waves of novel corticotroph and gonadotroph cells after adrenalectomy or gonadectomy (19, 20).

Putative mouse pituitary progenitors express the transcription factor Sox2 and are mainly localized dispersed within anterior pituitary parenchyma and in the marginal zone, a remnant of Rathke's cleft. Functionally, they grow *in vitro* as spheroids and are able to differentiate into all hormone-secretory cell types (21, 22). The major role of Sox2-expressing cells in adult mouse pituitary regeneration and plasticity has been further strengthened by the observation that these cells self-renew and naturally give rise to secretory cells throughout organism lifespan (23–25), even in response to selective ablation of mature endocrine pituitary cells (23, 26). Sox2 expression has been often associated with Sox9 and CD133 expression, as well as with the activation of developmental pathways, essential for stem cell homeostasis and embryogenesis. Although most studies have been carried out using adult mouse pituitaries, the similarity of the pattern of SOX2 expression in human pituitary, and the close structural resemblance of the stem niche from rodents to humans, allowed the extrapolation of the information from the animal model to humans (27).

Undoubtedly, multiple human stem cell populations depend on SOX2 activity for persistence and differentiation. However, in adult rat or mouse pituitary besides Sox2-expression, cells positive for Sox9, Nestin (28, 29), S100 (30, 31), E-cadherin, Oct4, Prophet of PIT1 (Prop1) (32, 33), Paired-Related Homeodomain Proteins 1 and 2 (Prrx1/2) (34–36), and glial cell line-derived neurotropic factor receptor-α 2 (GFRα2) (32, 35, 37), have been proposed as stem/progenitor candidates, with the expression of the different markers sometime overlapping within the same cell subset. Currently, only fragmentary evidence of stem-like properties of these cell subpopulations is available, being occasionally tested for the ability to undergo epithelial-mesenchymal transition (EMT) (38), to form colonies (13), and to retain multipotency (39). In rats, post-natal pituitary stem/progenitor cells were detected in the marginal zone and/or identified as GFRα2^+^/Sox2^+^/Sox9^+^ cell clusters scattered within the parenchyma of anterior pituitary; hormonal modulation at the middle of gestation increases the number of cells expressing stem markers in the marginal zone, while at beginning of lactation differentiated markers were predominant in the parenchyma and correlated to changes in cell proliferation; this observation supports the hypothesis that the PSC niche actively drives physiological pituitary plasticity (40).

Among embryonic transcription factors correlated to stemness, Prop1 has been detected in adult PSCs to regulate EMT (32, 33, 38). In rat anterior pituitary, hormonal cell differentiation is associated with Prop1 and Sox2 downregulation during post-natal development (21, 29, 33, 41, 42). In addition, SOX2 and PROP1 double positive cells, also expressing embryonic PRRX1 and PRRX2, have been detected in adult human pituitary gland (34). Mouse Sox2/Prop1-expressing PSCs are characterized by the co-expression of GFRα2, β-catenin, E-cadherin, as well Sox9, and Oct4, spherogenesis ability *in vitro*, and low proliferative rate *in vivo* after birth, suggesting their undifferentiated, stem-like nature (32).

As observed for stem cells from different tissue, Sox2^+^/Sox9^+^/E-cadherin^+^ PSCs have been detected within specific niches, which in adult mouse and rat anterior pituitary were identified in the marginal zone (21, 29, 32, 39, 41), and within the anterior lobe parenchyma (21, 25, 42–44). A similar organization and expression profile of PSCs has been described in the human gland (27, 32), although their regulation, likely involving soluble factors, cell surface proteins and extracellular matrices, is still not completely clear (31, 42, 44–47).

Among gene and protein signatures of the cells populating stem cell niches, the chemokine CXCL12 and its receptor CXCR4 are commonly detected. CXCR4 has been identified in different adult organs, including neuroendocrine tissues; in particular this receptor/ligand system is expressed in human normal anterior pituitary (48, 49), in both hormone-secreting (50) and non-hormonal cell types of humans and rodents (i.e., FS cells) (16, 51, 52). Post-natal mouse PSCs, isolated as side population (SP) cells in flow cytometry experiments, show Cxcl12/Cxcr4 expression (29) as well as S100-positive cells, likely comprising the Sox2^+^-stem/progenitor cells detected in the rat anterior lobe (41, 52). CXCL12/CXCR4 chemokine system is crucial for CNS development, functioning, and stemness maintenance (53), being expressed in embryonic and adult CNS stem cells, and playing a role in pituitary stem-related plasticity (54). *CXCR4* is upregulated in putative PSCs (44, 52), and SP cells of mouse pituitary gland (34). In the stem cell niches CXCL12/CXCR4 axis acts as chemoattractant and trophic factor for several cell types *via* paracrine and/or autocrine mechanisms (55), and induces EMT in progenitors (47, 56); this activity was also described in pituitary (51), further suggesting an association between its expression/activity and the stem cell phenotype.

Moreover, tissue/organ regeneration after physical or immunological injuries implies the activation of PSCs (57, 58) as described in pathological conditions which frequently cause transient or permanent hypopituitarism (e.g., altered embryonic formation, traumatic brain insults, tumor growth, or resection) (59). Since these clinical conditions constrain patients to a lifetime hormone replacement, a better understanding of the PSC regenerative potential and the mechanisms involved, could represent a therapeutic option for hypopituitarism. Transgenic mouse models were used to induce ablation of specific pituitary hormonal lineages to mimic pituitary injuries and study the regenerative properties of PSCs (60). For example, in *GHCre/iDTR* mice, diphtheria toxin (DT) treatment causes the elimination of GH-secreting cells. These experimental conditions trigger the activation of Sox2^+^ cells giving origin to Sox2^+^/GH^+^ cell population (23), although this regenerative capacity of the pituitary is time- and age-limited (58). Similarly, the differentiation potential of PSCs was reported after lactotroph ablation (23). Conversely, using an ACTH-secreting cell ablation model, authors reported that self-duplication of residual mature cells, rather than PSCs, is the predominant source for corticotroph restoration/replacement in the adult (61).

More recently, *in vitro* 3D multicellular structures (organoids), which better recapitulate phenotype and functions of the original tissue (62), have been proposed as a new experimental way to investigate PSC biology and differentiation pathways upon injury-activated stimuli (63). Organoid cells, derived from normal and GH-depleted adult mouse pituitary, mainly express Sox2 and E-cadherin, as previously described for PSCs (21, 32) and retain Sox2 after expansion in culture, showing limited differentiation capacity. Organoids from cell cultures of injured *GHCre/iDTR* pituitary show cystic structure, low proliferative activity, immature pituitary phenotype and alterations in specific pathways (i.e., Wnt/Lgr) as compared to dense undamaged pituitary organoid models, thus representing a valuable tool to study the regulation of putative PSCs in both normal and activated conditions (63).

**Figure 1:**
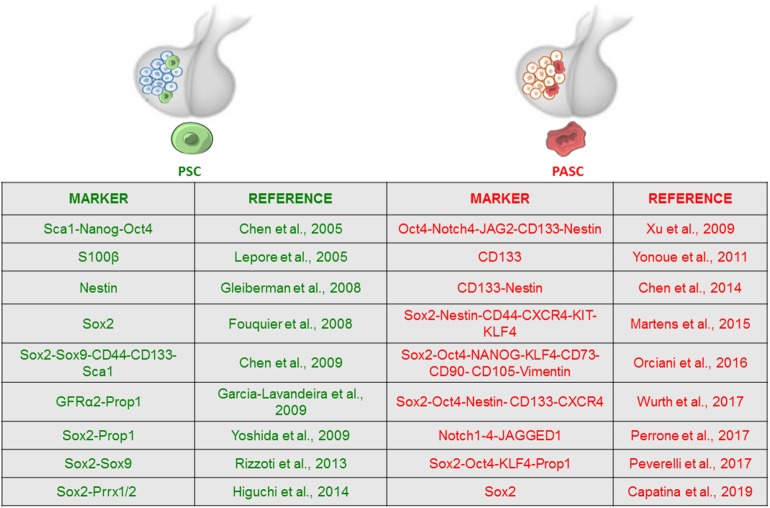
Expression of stem cell-associated markers in adult pituitary stem cell (PSC) and pituitary adenoma stem cell (PASC) populations. Adult PSCs exist within normal anterior pituitary and have been characterized by the expression of a discrete number of stem cell markers, most of them showing overlapping expression with Sox2-positive cell subset. Evidence supporting the presence of pituitary tumor stem cells is also based on enhanced stem cell marker expression. The figure summarizes the main markers used to identify the stem cell phenotype and corresponding references.

Overall, Sox2^+^ cells, persisting throughout life and being able to differentiate into pituitary hormone-secreting lineages, represent the most widely validated *in vitro* PSC model; however, studies performed in different animal models did not report a univocal phenotype, and the existence of a single or distinct stem cell populations is not definitely proven. Therefore, the expression of multiple markers (Gfrα2-3, Prop1, Sox2, Oct4, Sox9, β-catenin, E-cadherin), outlined in Figure 1, might reflect *in vivo* heterogeneity of both PSCs and committed progenitor populations, indicating the existence of cell subsets in the pituitary, different for origin, phenotype, activation of transcription factors, or niche interactions, which display different functions in adult human organ plasticity. Further characterization of human adult PSCs will allow a better understanding of the physiological and pathological roles of these cell subsets.

## Cancer Stem Cells in Pituitary Adenoma

A detailed characterization of CSC-like subpopulations was performed in the adamantinomatous craniopharyngioma, a low-grade pediatric pituitary tumor originating from Rathke's pouch, which may display an aggressive clinical course (64). Moreover, the CSC hierarchical tumorigenesis model was also proposed to be at the basis of the development of other benign tumors, and, in particular, pituitary adenomas (PAs) (54, 65, 66). As performed in other tumors, identification and isolation of PA stem cells (PASCs) relies on immunostaining followed by FACS or image microscopy (immunohistochemistry or immunofluorescence) in order to detect surface stem markers in post-surgical pathology preparations or, more recently, in *in vitro* cell cultures enriched in stem-like subpopulations by flow cytometry or growth in stem cell permissive media. However, CSCs are mainly operationally defined, using functional assays such as assessment of cell proliferation or clonogenic activity (to detect sustained proliferative potential) and sphere formation assay (as an index of self-renewal ability), Nonetheless, since *in vivo* tumorigenic property still represents the main defining parameter for CSCs, mouse xenograft is the most reliable method for assessing the presence of CSCs in a cell culture. However, in PAs, cell senescence, involved in preventing the malignant features in these tumors, is believed to inhibit the development of PAs in mouse models (67), and indeed not all the studies were able to demonstrate this feature in mice, leading to the establishment of alternative animal models, i.e., tumor growth in zebrafish embryos (68).

The following paragraphs will highlight consensus and diverging reports on these features in PASCs and the implications for their definition. A comparative analysis of the data from the different studies are summarized in Table 1.

**Table 1:** Isolation and functional characterization of pituitary adenoma stem cells.

	**References**	**Xu et al. (69)**	**Yunoue et al. (70)**	**Chen et al. (71)**	**Donangelo et al. (72)**	**Mertens et al. (73)**	**Orciani et al. (74, 75)**	**Megnis et al. (76)**	**Wurth et al. (77)**	**Peverelli et al. (78)**
Experimental model and procedure	PA type	GH-oma, NFPA	GH-/ACTH-/ TSH-/PRL-oma, NFPA	Unknown	Spontaneous PA from Rb*^+/−^ mice*	GH-/ACTH-/PRL-oma, NFPA	GH-oma, NFPA	GH-/LH-oma, NFPA	GH-/ACTH-/ GH-PRL-oma, NFPA	NFPA
	Model	HUMAN	HUMAN	HUMAN	MOUSE	HUMAN	HUMAN	HUMAN	HUMAN	HUMAN
	Isolation approach	Floating spheres, serum-free medium	CD133^+^cell-identification by FACS analysis	Serum-free medium-	Floating spheres, serum-free medium	SP, serum-free medium	Cell adhesion, MSC-medium	Cell adhesion, serum-containing medium	Serum-free medium and CD133^+^ cell sorting	Serum-free medium
Cancer stem cell criteria	Spherogenesis (self-renewal)	Yes	n.d.	Yes	Yes	Yes	No	n.d.	Yes	Yes
	Multipotency (differentiation)	Yes (hormone-secreting cells)	n.d.	Yes (neural lineages)	Yes (hormone-secreting cells)	n.d.	Yes (mesenchymal lineages)	Yes (mesenchymal lineages)	Yes (hormone-secreting cells)	n.d.
	*In vivo* tumorigenesis (animal model)	Yes (M)	n.d.	Yes (M)	Yes (M)	No	n.d.	n.d.	Yes (Z)	Yes (Z)
	Other features	Cytotoxic drug resistance	-	Low proliferative activity *in vitro*	-	-	SSTR1-5 expression, sensitivity to anti-proliferative effects of SSTR agonists	SSTR1-5, D2R expression (low)	SSTR2, SSTR5, and D2R expression; sensitivity to anti-proliferative effects of D2R/SSTR chimeric agonist	SSTR2 and D2R expression, sensitivity to antiproliferative effects of SSTR2 and D2R agonists

*PA, pituitary adenoma; M, mouse; Z, zebrafish embryo; SP, side population; MSC, mesenchymal stem cell; SSTR, somatostatin receptor, D2R, dopamine receptor2; serum-free medium, stem cell permissive medium with EGF and bFGF; n.d., not determined*.

### Isolation of Putative Pituitary Adenoma Stem Cells

Most reports describe the *in vitro* isolation of putative PASCs through the subpopulation selection using stem cell-permissive media originally used to isolate and cultivate neural stem cells. In particular, beside few differences, a common, fundamental feature is observed in all media formulations from the published studies: the absence of serum and the presence of growth factors, generally EGF and bFGF. Serum-free medium was already used to enrich in CSCs, cultures from several different solid tumors (79) and allowed to retain *in vitro* genotype and phenotype features similar to the original tumor (80). In almost all the studies to date published, these experimental conditions held to isolate PA cells able to grow in suspension as spheroids, a feature generally considered an *in vitro* index of self-renewal.

However, it has to be remarked that, to date, the success rate of this procedure is still far from 100%. In the study of Xu et al. two out of eight of the tested PAs gave rise to stem-like cell cultures (69). In another study, 69.6% of cultured non-functioning PAs (NFPAs) (*n* = 46) showed the formation of non-adherent spheroids after 2 weeks of culture in stem cell-permissive medium; sphere generation was significantly higher in aggressive tumors as assessed by cavernous sinus invasion (78). In our lab, we developed a similar protocol to isolate and expand *in vitro* putative PASCs from GH-secreting PAs (GHomas) and NFPAs post-surgical specimens (81), obtaining a similar success rate. In particular, 68% of the PAs (*n* = 38) were able to proliferate as spheroids when selected in stem-cell permissive medium (77). Importantly, in this study we demonstrated that PASC selection, performed either by growth in stem cell-permissive medium or by sorting for CD133 expression, gives rise to cell subpopulations endowed with comparable stem cell-like features; these results highlight the relevance of CD133 in PASCs, as also reported in CSCs from different malignant tumors (82). A similar approach was described by Zhao et al. who sorted CD133^+^ and NESTIN^+^ co-expressing cells from dispersed PA cells. These cells represented up to the 3% of total PA cells and were able to generate spheres for several passages (83). In other reports, stem cell-permissive medium allowed the selection of sphere-forming cells in cultures from all the 12 (71) or 14 (84) tumor analyzed including GH-, ACTH-, FSH/PRL-secreting PAs and NFPAs. Similar results were obtained in a mouse model (*Rb*^+/−^ mice), which spontaneously develops PAs: the growth of explanted tumor cells in stem- permissive culture conditions held the isolation of sphere-forming putative PASCs (72). A stem-like cell subpopulation was also derived from three human GHomas and three NFPAs, growing dispersed cells using culture conditions developed for mesenchymal stem cell (MSC) cultures, although these PASCs showed peculiar characteristics as compared to the others studies (see below) (74).

A different approach was used in another study (73), in which the same methodology used to isolate adult PSCs from normal murine pituitaries was applied (85). Since one of the typical features of cells with a stem-like signature is the overexpression of ATP-binding cassette (ABC) multidrug transporters, which confers resistance against toxic stimuli (included those exerted by drugs), they analyze PA cultures for the ability to extrude, via these transporters, the fluorescent DNA-binding dye Hoechst 33342, appearing in the FACS analysis as a “side population” (SP). Interestingly, SP cells were isolated form all the 60 tested adenomas (GH- and ACTH-secreting, or NFPAs), with a calculated presence of putative PASCs ranging from 0.5 to 2% (mean 1.9%), with the occasional observation of a NFPA showing up to 17.2%. SP cells grew as spheroids *in vitro*, even though only short term proliferation was observed, and, to perform a more detailed characterization of stem properties, SP cells derived from the established murine corticotroph adenoma cell line AtT20 were analyzed (73).

### Stem Cell Markers and Intracellular Pathways in Pituitary Adenoma Cells

Although tissue-specific transcription factors can be identified in normal stem cells from different tissues, normal stem cells are generally defined by the expression of a common subset of stemness-related factors (e.g., SOX2, NANOG, OCT4, NOTCH, CXCR4/CXCL12, CD44, etc.) which confers the peculiar properties of self-renewal and pluripotency, during both embryonic development and adult stage (13). Candidate PASCs also display expression of several markers used for stem cell identification by immunophenotyping. For example, SOX2 and NANOG are two pluripotency-associated transcription factors expressed by embryonic and adult stem cells, involved the maintenance of stem cells in various adult tissues, and, in mouse, Sox2^+^ cells are involved in pituitary regeneration (26). NOTCH signaling contributes to stem cell proliferation (86) and prevents progenitor cells from premature differentiation (87), while OCT4, essential during embryogenesis and to retain pluripotency in the adult tissues, is overexpressed in CSCs of various cancers conferring drug resistance (88). CD133 (prominin 1) is a membrane glycoprotein involved in retinal development, whose overexpression is related to aggressiveness of ovarian, colorectal, prostate, and lung cancer, and glioblastoma, representing a signature for putative CSCs in these neoplasms (89); the chemokine CXCL12 and its receptors CXCR4/CXCR7 are involved in self-renewal and migratory behavior of normal stem cells and CSCs (90, 91) acting via autocrine/paracrine mechanisms (52, 55). Importantly, SOX2, NESTIN, GFRα2, among others were established as markers to identify normal PSCs and progenitors (92). For example, SOX2 and OCT4 expression was detected in scattered cells within human normal pituitary samples, while more diffuse expression was observed for CXCR4 (77).

The evaluation of the expression pattern of above stem markers, summarized in Figure 1, represents the basis for the characterization of CSC isolated from PAs.

#### Stem Cell Markers in Histological Pituitary Adenoma Preparations

The first evidence of the presence of putative stem-like cells within PAs was obtained from histological preparations of human tissues. CD133 was analyzed in a series of 70 PAs, observing that 25.7 % of them showed positive immunolabeling, with higher frequency in NFPAs as compared to GHomas (15/45 in NFPAs vs. 3/25 in GHomas and prolactinomas) (70). After cell dispersion and cytofluorimetric analysis, CD133-positive cells were shown to represent an average of 2.9% of total adenoma cells in NFPAs (*n* = 5), 0.8% in GHomas (*n* = 2) and 7% in the single prolactinoma analyzed. However, CD133 expression levels were not correlated with tumor size, or post-operative recurrence rate. CD133^+^ cells also co-expressed NESTIN and, although with high intertumor variability, CD34. However, no statistically significant correlation between CD34 and CD133 expression was observed. Wurth et al. identified, by immunofluorescence, the expression of SOX2, OCT4, NESTIN, and CD133, in seven human GHoma, and five NFPA samples, while NANOG and NOTCH1 were detected in 25 and 50%, respectively, of the samples analyzed; these markers restricted to subsets of cells diffuse within tumor mass, but, in GHomas, do not co-localized with GH-secreting cells (77). Subsequently, small cell subpopulations expressing CD133 and NESTIN were identified in 12 human prolactinomas by immunofluorescence analysis. Interestingly, CD133 and dopamine D2 receptor (D2R) expression, analyzed by FACS, segregate in different subpopulations (93). In another series of human PAs, NOTCH 1–4 receptor subtypes, and their ligand JAGGED1 were detected by RT-PCR, suggesting the presence of a constitutive autocrine/paracrine NOTCH system activation in a subset of tumor cells (94). In this study, NOTCH3 immunohistochemistry showed that NFPAs, prolactinomas, GHomas, and ACTH-secreting PAs (ACTHomas) express the protein in the cytoplasm and membrane of tumor cells. SOX2 expression was also detected by immunohistochemistry in about 50% of 16 PAs, with prevalent expression in GHomas and prolactinomas (60% of cases) than in NFPAs (only 20% of cases) (95). Transcriptomic analysis confirmed an increased expression of NOTCH3 in human NFPAs compared to normal pituitaries (96), as further demonstrated by other studies at both mRNA and protein levels (97–99).

In a large cohort of 65 PA specimens, *CXCR4* mRNA was detected in 92% of GHomas and 81% of NFPAs, whereas the ligand *CXCL12* was identified in 63 and 78% GHomas and NFPAs, respectively. These data were confirmed by immunohistochemistry and immunofluorescence, in GHomas and NFPAs showing higher level of expression than normal human pituitary samples (48). Flow cytometry and immunofluorescence analysis of 35 PAs (21 invasive and 14 non-invasive) also revealed that CXCR4 and CXCL12 expression was significantly higher in the invasive subset than that of the non-invasive PAs (100); conversely no correlation was observed for other markers such as CD44 and CD147. Interestingly, CXCR4 activation in the rat somato-prolactinoma GH4C1 cells enhances proliferation and hormone release (101), suggesting a pivotal role for this chemokine system in PA functioning.

#### Pituitary Adenoma Stem Cell Markers in Enriched *in vitro* Cultures

**Figure 2:**
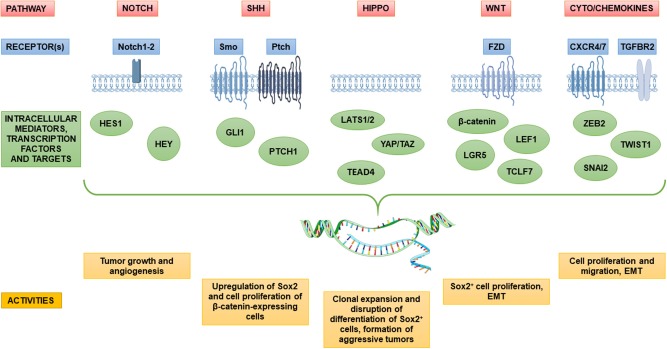
Dysregulation of major stem cell pathways in candidate pituitary adenoma stem cells and their involvement in pituitary tumorigenesis. The diagram depicts key signaling pathways in pituitary adenoma implicated in sustaining the putative pituitary adenoma stem cell populations and its functions in tumor maintenance and progression. Several pathways including Notch, Sonic Hedgehog (SHh), Hippo and Wnt, which are tightly regulated in normal stem and precursor pituitary cells, are aberrantly regulated in tumors, likely in defined subsets of cancer stem cells within pituitary adenomas. Signaling pathways are often linked to epithelial–mesenchymal transition (EMT) in the gain of tumor stem cell properties, and migration ability. An overview of main receptors and transcriptional mediators upregulated in pituitary adenomas is represented as well as significant effects on the pathogenesis of pituitary adenoma.

One of the first study reporting the phenotypical characterization of putative stem cells isolated by two PA cell cultures (one from a GHoma and one from a NFPA) was performed by Xu et al. (69). They obtained pituitary cell spheroids which express OCT4, NOTCH4, JAG2, CD133, and NESTIN. Subsequently, similar or partially overlapping results were obtained in many different studies in which PASCs were obtained with different experimental approaches. In detail, a second study showed that sphere colonies derived from PAs express CD133, NESTIN and other stem markers typical of neural cells: NCAM (neural cell adhesion molecule) and neuron-specific class II β-tubulin (71). Moreover, in agreement with previous studies, PASCs isolated as SP, besides NESTIN, revealed the expression of other stem cell-related markers, such as CD44, CXCR4, KIT, KLF4, and SOX2 (73). Importantly, EMT-related gene expression, such as *TWIST, ZEB1* and *2*, and *SNAI1* and *2* was up-regulated in candidate PASCs, as compared to non-SP cells, while the epithelial marker genes *CDH1* and *CLDN1* were down-regulated (73), indicating that EMT is active in PASCs. The highest relative levels of some EMT-associated genes (*ZEB2, SNAI2*, and *TWIST1*) occurred in the SP derived from invasive PAs, further highlighting the clinical implication of these regulatory pathways in PASCs (Figure 2). However, the EMT activation in PASCs will require further validation.

In agreement with these results, our group reported that PASC-derived pituispheres express SOX2, OCT4, NESTIN, CD133, NOTCH1, and CXCR4 in almost all the culture analyzed (93, 100, 87, 91, and 100%, respectively), while NANOG expression was confined to only half of the PAs (77). A partially divergent result was reported by Orciani et al. in a subset of six GHomas and six NFPAs, in which, together with the expression of pluripotency factors (OCT4, NANOG, KLF4, CD133, and SOX2), the typical MSC surface markers were identified in PASCs (74). Indeed, these cells were isolated using MSC-permissive culture medium and, in appropriate culture conditions, they were able to differentiate into osteogenic, lipogenic, and chondrogenic cell lineages. Subsequently, using similar cultures from different tumors, these Authors reported that PASCs also express markers reminiscent of EMT (75). Similarly, in a different study, PASCs isolated from two GHomas, three prolactinomas, and two NFPAs displayed MSC features, as far as morphology, cell surface marker expression (CD73, CD90, CD105, CD44, and vimentin), and differentiating ability into osteocytes and adipocytes (76). These results, in line with reports on better characterized malignant tumor CSCs, i.e., glioblastoma (102), suggest the existence of different CSC subpopulations within PAs, characterized by different identities and, possibly, functions and origin within adult pituitary, although partially sharing pluripotency markers.

In another report (78), putative PASCs were isolated from 46 NFPAs and characterized for the expression of *SOX2, OCT4*, and *KLF4* mRNAs. SOX2 and NESTIN expression was also confirmed by immunohistochemistry/immunofluorescence experiments, in a subset of PASC-enriched cultures grown as spheroids. Moreover, indirect immunofluorescence analysis revealed the nuclear co-expression of the pituitary specific transcription factor PROP1 and SOX2 in about 2–10% of cells, while PROP1 was cytoplasmic in SOX2^−^ cells, highlighting the presence, within the culture, of heterogeneous populations of stem (PROP1^+^/SOX2^+^/) and precursor (PROP1^+^/SOX2^−^) cells Finally, the analysis of cells from 22 PAs of different subtypes showed a preferential high expression of CD15, as compared to CD133. Moreover, by comparative analysis within each tumor cell culture, of CD15^high^ and CD15^low^ subpopulations, it was reported that CD15^high^ cells are also associated to higher *SOX2* and *PAX7* expression, and were detected in higher percentage in recurrent PAs as compared to the matched primary tumor (84).

To date only one study formally described the *in vitro* isolation of murine PASC, spontaneously originated from pituitary intermediate lobe in *Rb*^+/−^ mice. Isolated cells were grown as spheroids and expressed Sox2, Nestin and CD133, as well as Sca1, in line with what reported in normal PSCs (72). Nevertheless, this study is particularly important since it implies a common phenotype of PASCs, independent from the human or murine origin.

#### Stem Cell-Related Pathway Activity in Pituitary Adenoma Cell Cultures

Altogether, previous described literature supports the alteration of stem cell regulatory pathways in cell subpopulations within PAs. Among these, the dysregulation of NOTCH pathway was associated with PA, being gene expression of components of its signaling (e.g., *HES1, HEY, NOTCH1, NOTCH2*) upregulated in the SP from human PA (73). However, functional data able to clarify its role in PASC tumorigenesis are very limited, and mainly obtained in established PA cell lines. To address this issue, Zubeldía-Brenner et al. (103) used the rat somato-prolactinoma cell line GH3 [previously showed be positive for Notch1-4 (94)] to determine whether the inhibition of Notch signaling would affect growth and angiogenesis of tumor xenografts in immune-compromised mice. For this purpose, they used a γ-secretase inhibitor (DAPT), which prevents cleavage of intracellular Notch domains and, consequently, modifies target specific transcription factors in the nucleus. Interestingly, the inhibition of Notch signaling led to tumor mass reduction and decreased neoangiogenesis. In order to elucidate whether putative PASCs with constitutive active NOTCH system could be targeted by inhibition of this pathway, a significant research work in tumor subtype-specific manner has still to be done. Nevertheless, these recent data may support the downregulation of NOTCH signaling as a potential therapeutic tool in aggressive or resistant PAs.

Other studies identified a relationship between the expression and/or activity of different specific stem cell markers in pituitary tumorigenesis. The activity of SONIC HEDGEHOG (SHH) pathway, crucial during embryogenesis and organ development, differs among PA subtypes, being downregulated in NFPAs as compared to normal pituitary tissues, while GLI1, one of the transcriptional effectors that mediate SHH signal transduction, is overexpressed in GHomas and ACTHomas (73, 104). Excess Shh signaling increases both the proliferation of Sox2^+^ and Sox9^+^ adult mouse PSCs and the expression of ACTH, GH and PRL in adult pituitary (105). SHH and its target gene *GLI1* were also shown to be overexpressed in GHomas, ACTHomas, and prolactinomas, further supporting that excess SHH signaling is involved in the development/maintenance of hormone-producing PAs. SHH administration to the HP75 human PA cells caused the upregulation of SOX2 and proliferation of β-catenin-expressing cells. Similarly, the addition of WNT3a in SOX2-expressing PA cells sustains cell proliferation. It was proposed that SOX2 mediates crosstalk between SHH and WNT/β-catenin pathways to promote the proliferation of PAs (106). In fact, aberrant WNT/β-catenin activity has been observed in pituitary tumors (107), and gene expression of components of the pathway (*LEF1, LGR5, TCF7L1*) was identified in PA SP cells (73).

The HIPPO kinase cascade is a crucial pathway that regulates cell growth and fate in numerous organs, and may be implicated in the regenerative response of PSCs (58). In mouse PSCs, this signaling activity, in association with its effectors Yes-associated protein (Yap) and transcriptional co-activator with PDZ binding motif (Taz), was detected in Sox2-expressing subpopulations. Moreover, overexpression of HIPPO pathway components (i.e., large tumor suppressor homolog 2, LATS2, and TEAD4), has been described in PA SP cells (73) as well as in poorly differentiated pituitary tumors (null-cell PAs, adamantinous and papillary craniopharyngiomas) which display enhanced YAP/TAZ activity (108). Importantly, the activation of HIPPO pathway, by silencing the kinase LATS1, inhibits promoter activity of *GH1* and *PRL* genes, correlating high YAP/TAZ activity with repression of pituitary differentiation (108). Using a genetic approach to perform gain- and loss-of-function experiments in mice, it was shown that *Yap/Taz* activation during development is essential for normal pituitary formation from Sox2^+^ PSCs. However, postnatal upregulation of Yap/Taz activity causes uncontrolled clonal expansion of Sox2^+^ pituitary cells, disruption of their differentiation, and formation of aggressive NFPAs, highlighting that this regulatory signaling cascade is a relevant component for both pituitary development and tumorigenesis (109).

Figure 2 summarizes the signaling pathways and transcriptional networks involved in PASC maintenance and tumorigenesis.

### Assessment of Biological and Functional Activity in Putative Pituitary Adenoma Stem Cells

As highlighted before, CSCs are not a single population within a given tumor, but they set apart into subpopulations characterized by heterogeneous phenotypes (110, 111). Thus, beside marker expression, the characterization of these cells requires the establishment of biological and/or functional specific activities. In particular, by definition, CSCs should display: (i) self-renewal, mainly assessed by serial sphere-forming ability, (ii) long term proliferation *in vitro*, and (iii) capacity to differentiate into specific cell types related to the tissue of origin.

#### *In vitro* Self-Renewal Activity

*In vitro* spherogenesis is a commonly accepted index of the self-renewal ability of a cell population. Although all the studies addressing the role of PASCs as PA-initiating cells, used the ability to form pituispheres to identify these subpopulations, in order to validate the CSC phenotype, this ability should be a persistent feature of the isolated cells. This can be assessed measuring the generation of secondary (tertiary, or more) spheres after disaggregation of the initially generated spheroids. This ability was indeed described in most of the previously mentioned studies: Xu et al. reported that after up to 15 passages PASCs were still able to generate spheroids (69); Chen et al. observed that, after dispersion of PAs to single cells, primary spheres are generated after 2–3 weeks in culture and that daughter cells derived from the spheres formed secondary spheres within 2–4 weeks after reseeding (71); Peverelli et al. showed that cells from 46 PAs form primary spheres after about 2 weeks in stem cell-permissive medium, with sphere-forming efficiency (SFE), calculated in a subset of tumors, in about 2–5% of the plated cells. Cells derived from primary spheres and re-plated as single cell were able to generate secondary spheres with a SFE of about 4–8%, while no tertiary spheres were obtained (78). In our study, sphere formation occurred after 7–10 days in 14 out of 16 GHoma and NFPA cell cultures, but proliferation within spheroids lasted for several weeks (77). In a subset of samples in which it was calculated, SFE was in the range of 1.3–7%. Cells harvested from disaggregation of 7 days living spheroids were able to generate secondary spheres within 13 days culture in stem cell-permissive medium, with a slightly higher SFE in secondary spheres as compared to that of the primary spheroids derived from the same PA (77). Similar data were obtained in PA cultures derived from the *Rb*^+/−^ mice: spheres were generated starting after 3 days *in vitro* and continued to grow up to 10–12 days. After dissociation, although the number of viable cells declined, cells from primary spheres generated secondary spheres displaying similar growth rate, for up to 12 passages. As compared to non-stem population (identified by the lack of Sca1 expression), PASCs showed four-time higher propensity to generate spheroids (72).

Spheres also developed from SP cells isolated after dispersion of PA tissues, while the main population was devoid of such activity. However, SFE was very low, with only 2–4 per 20,000 cells seeded, which did not allow to have viable cells in a sufficient number to test spherogenesis in further passages (73).

#### Proliferation and Migration Abilities

Primary cultures of non-stem PA cells have a very low proliferative activity, with no more than 1–2 *in vitro* cell divisions (112, 113). Thus, sustained and long-lasting proliferation *in vitro* may represent a relevant index to identify cell subpopulations endowed with stem-like characteristics. However, only few studies analyzed this feature: Wurth et al. reported that cells derived from 11 PASC-enriched cultures proliferate up to 28 days increasing up to 400% the initial number, although for longer culturing time the proliferative potential declines. Conversely, non-stem PA cell cultures barely duplicated once, and started to degenerate from the second week *in vitro* (77). Peverelli et al. reported that NFPA PASC spheroids contain a high percentage of Ki67^+^ cells, which lasted up to 4 weeks, while the differentiated cell counterpart retains a proliferative activity for no longer than 1 week (78). Similarly, stem-like cells derived from *Rb*^+/−^ mice PAs, characterized for Sca1 expression, exhibit a proliferative advantage over Sca1^−^ cells, evaluated up to 3 days in culture and reaching a 4.4-fold increase in growth rate (72). Orciani et al. reported a linear growth of mesenchymal PASCs for up to 12 days, with a doubling time of about 20 h for the first eight passages (74). However, at the 12th *in vitro* passage the doubling time reached 40 h, further confirming, that PASCs tend to lose the stem cell-like features, as observed in different *in vitro* models (77). Megnis et al. reported a continuous proliferation in all the 6 PASC-enriched cultures analyzed, up to 14 days (76). Other studies were not able to observe a sustained *in vitro* proliferation and did not analyze this parameter (73). The limited *in vitro* growth of PASCs, although much more prolonged as compared to non-stem PA cells, might either reflect intrinsic features or technical limitations in the cultivation of these cells. In this respect, the benign nature of PAs, in which the activation of senescence program limits tumor expansion, could determine the self-limiting growth of these cells *in vitro*. Moreover, it should be considered that culture conditions for PASCs are mainly adapted from CSC cultures of different tumors (79), and may be inappropriate or insufficient to support their continuous growth. Further studies are required to address this issue.

CD133^+^ cells isolated from both GHomas and NFPAs also possess a higher migratory activity as compared to CD133^−^ cells isolated from the same tumor (74). This observation provides a direct support to the correlation previously reported of a higher number of CD133^+^ cells in invasive PAs as compared to those with a non-invasive clinical behavior (71).

#### Differentiation Ability

Stem cell are undifferentiated cells acting as a reservoir from which differentiated cells origin to replace cells during normal adult tissue turnover or after injuries. This feature is also present in CSCs which are the source of all the “differentiated” tumor cells forming the bulk of the tumor. In several tumors CSCs possess a marked *in vitro* differentiation ability. For example, glioblastoma CSCs, grown in the absence of growth factors and in the presence of fetal bovine serum (FBS), are able to differentiate into astrocyte- and/or neuron-like cells (114). Contrasting results were reported as far as PASC differentiation ability: in most studies this PA subpopulation does not express pituitary hormones (69, 70, 77), while, in others, the immunohistochemical detection of LH in Sox2^+^ cells within spheroids was reported, although evidence of co-localization in the same cells was not provided (78). However, relevant to the CSC ability to originate all the cell populations composing the tumor, few studies actually showed that PASCs, isolated from hormone-producing tumors, can differentiate into hormone-releasing cells *in vitro*. Xu et al. showed, using specific ELISAs, that when shifted in differentiation medium (in which growth factors were replaced by FBS) for 2 weeks, PASCs derived from a GHoma released GH in response to GRF, and LH and FSH when exposed to LHRH (69). This was not observed in the same cells grown in stem cell permissive medium. However, both stem-like and differentiated cells released PRL in response to PRL-releasing peptide, and TSH in response to TRH. NFPA PASCs also released LH in basal conditions, as also demonstrated by Peverelli et al. (78), and, after differentiation, released both LH and FSH after LHRH treatment. Thus, PASC differentiation potential was demonstrated, but it was still unaddressed the lack of a tumor-specific differentiation, since LH, FSH, and TSH were also produced by GH-secreting PA culture. PASC ability of to differentiate into cells with an adult pituitary cell phenotype was tested evaluating GH production in 2 GHoma PASC cultures after differentiation induced by shifting the cells in FBS-containing medium for 10 days (77). In these experimental conditions GH immunoreactivity, undetectable in the cells kept in stem cell-permissive medium, was clearly evident. Interestingly in the same cells the expression of some stem cell markers (CD133, OCT4, NESTIN) was reduced after differentiation, while others were retained (i.e., CXCR4). These data clearly support the differentiation potential of these PASC cultures, in which the loss of stemness markers is associated to the induction of hormone production. However, when cells were grown for 30 days in stem-permissive medium, few GH-expressing cells were observed also in these conditions, although in a lower number than observed after differentiation (77). Thus, a low level of spontaneous differentiation may occur in the PASC subpopulation, in line with the loss of proliferative activity after prolonged *in vitro* culture (see above). Similarly, PASCs, isolated from tumors developed in *Rb*^+/−^ mice and grown in the presence of growth factors, are completely hormone-negative, but after 9 days of culturing in differentiation medium all six pituitary hormones were detected in the cultures (72). Also in this experimental setting, stem cell markers Sox2 and Sca1 were downregulated, although few Nestin^+^ cells were observed in differentiated PASCs, sometime co-expressed with hormones (72).

Other studies reported the occurrence of heterologous type of differentiation. Differentiated PASCs were shown to express neural (β-III tubulin), astrocytic (glial fibrillary acidic protein, GFAP), and oligodendrocyte (2′,3′-cyclic nucleotide-3′-phosphodiesterase, CNPase) markers (69, 71).

On the other hand, when PASCs were selected according to MSC isolation procedures and characterized by the expression of mesenchymal markers, differentiation follows this lineage and not the pituitary-related phenotypes. In fact, shifting the cultures in defined media, cells acquire osteogenic [expression of alkaline phosphatase after 10 (74) or 21 (76) days], adipogenic (presence of cytoplasmic lipid vacuoles after 14/15 days) (74, 76), or chondrogenic (detection of safaranin-O staining after 30 days) (74) phenotypes.

These latter studies further highlight the possibility that different stem-like subpopulations may be present within PAs, which can be preferentially expanded *in vitro* according to the isolation procedure used. Thus, more in depth lineage studies are necessary to address the cellular origin of these subpopulations.

### *In vivo* Tumorigenic Ability

The ability to regenerate in animal models the tumor from which stem cells were isolated is still the main feature which defines CSCs (115). Thus, the tumorigenic ability is a required feature also for PASCs. However, when benign, slow-growing tumors are studied, the development of animal model is a critical issues and, to date, contradictory results were reported.

Several of the previously analyzed studies did not address this issue in the characterization of PASCs (70, 73, 74, 76), while others used immunodeficient mouse models to perform xenograft experiments, as commonly performed for CSCs from malignant tumors (glioblastoma, breast cancer, mesothelioma, osteosarcoma) (114, 116–119).

Xu et al. succeeded in inducing tumor development after brain parenchyma injection of 10,000 cells from both the two cultures of PASCs they isolated. Xenografts were slow-growing, being detectable after 3 months from the inoculation, but still expanding after 6 months (69). Interestingly, the nature of the original tumor was retained since tumors originated from GHoma-isolated PASCs showed expression of GH (and PRL), which was not observed when PASCs derived from NFPA were injected. Moreover, PASCs recovered from tumor xenografts were expanded *in vitro* as spheroids and re-injected in mouse brains, retaining tumorigenicity. Similarly, a small tumor mass was observed 12 weeks after subcutaneous (s.c.) inoculation of 10,000 cells from spheroid cells isolated from a single PA (71). The resulting single tumor displayed low proliferation rate (as for Ki67 labeling), histology resembling PA structure, and expressed synatpophysin, but it was not further characterized. Similar results were obtained by the intracranial injection in nude mice of 10,000 CD133^+^/nestin^+^ PA cells, which gave rise to tumors, detectable after 6 weeks from the graft (83). In another study, one PASC culture from a null PA, sorted for CD15 expression, was pseudo-orthotopically injected in the brain of three mice (84). In agreement with the CSC theory, 50,000 CD15^+^ cells generated tumors when injected in the mouse brain, while up to 500,000 CD15^−^ cells were not tumorigenic. The explanted tumors showed high number of mitosis and nuclear atypia, and, in agreement with the histological subtype of the non-secreting original tumor, immunoreactivity for pituitary hormones was not detected. Comparing the original tumor with the xenograft, both specimens were negative for GFAP, alpha-smooth muscle actin, EMA, and TTF1, and, according to the neuroendocrine derivation, both expressed synaptophysin and CD56. Interestingly, desmin and myogenin were unexpectedly expressed only in the xenograft but not in the original PA, suggesting that CD15^+^ PASCs possess a microenvironment-dependent multi-lineage potential (84).

Conversely, Mertens et al. failed to demonstrate tumorigenicity of PA cells *in vivo* by xeno-transplantation in immunodeficient mice, regardless their hormonal phenotype. In these experiments small tumor pieces were implanted s.c. or under the kidney capsule in different mouse models: SCID mice, or the more immunodeficient NOD–SCID and NOD-SCID IL2rγ^null^ (NSG) mice, which lack mature T, B, and natural killer cells. Tumor specimens apparently survived for 3–4 months after implantation but no growth was detected. Similar negative results were also obtained using xenografts of dissociated PA cells (73).

Also in our experience, we did not detect tumor development within 8 months from the injection of PA cells that fully accomplish the *in vitro* criteria for CSC definition (i.e., stem marker expression, undifferentiated phenotype and differentiation ability in hormone-producing cells, high proliferation potential, self-renewal); similar negative results were observed after grafting the cells either s.c. into the flank of animals in the presence of Matrigel (20,000 cells, from 2 PAs) or pseudo-orthotopically in the striatum (50,000 cells, from 4 PAs) (77). Thus, we used a different animal model: zebrafish embryos, which allow the evaluation of two relevant CSC features which define PASC tumorigenicity: invasive and angiogenic potential (120). PASCs from 5 PAs were xenografted into embryos of the Tg(*fli1:EGFP*)y1 zebrafish line that express EGFP in endothelial cells, thus allowing to detect whether neovessels are formed in proximity of the injected cells. PASCs were stained with a red fluorescent dye, to follow their migration activity. In this model, only a 500 cells/embryo are required and all the events occur within 2–3 days. Grafted PASCs were easily detected soon after the injection and, when recovered after 48 h, confirmed the human nature and CD133 expression. However, after 24 h injected PASCs start to migrate outside the tumor cell mass, invading the yolk and the caudal areas. Moreover, at odd with control embryos, grafted embryos showed the sprouting of new vessels from the subintestinal vein plexus, which were directed toward the tumor cells, demonstrating the induction of *in vivo* neo-angiogenesis. Similar results were also reported by Peverelli et al., who grafted sphere-derived cells from 2 NFPAs in zebrafish embryos. After 24 h, tumor cells from both tumors migrated outside the site of the injection confirming an invasive ability. Moreover, also in this study injected spheroid-derived cells stimulated the formation of endothelial sprouts from the subintestinal vessels plexus in 1/3 animals (78).

Finally, tumorigenic ability was also reported for PASCs derived from *Rb*^+/−^ mice, although this occurrence was somehow expected since they contain the specific genetic alteration responsible for the formation of these adenomas (72). In these experiments Sca1^+^ and Sca1^−^ cells were compared, demonstrating that the stem-like cell subpopulation was endowed with higher tumorigenic potential when injected in the striatum of NSG mice. Importantly, although injected cells were hormone-negative, after *in vivo* tumor development multiple hormones were detected in 91% of the 11 tumors analyzed. *Sca1* expression was abolished in almost all the tumors, whereas other stemness markers were retained (Nestin, CD133, Sox2) in subsets of cells, altogether with the astrocytic marker Gfap (72).

## Drug Sensitivity of Pituitary Adenoma Stem Cells

One of the most relevant information that can be obtained by identification of CSC from different tumors, is the possibility to analyze, with good translational potential, the sensitivity of this subpopulation to traditional or innovative pharmacological treatments. For example, studies on CSC from glioblastoma, breast cancer or osteosarcoma (119, 121–123), demonstrated a higher sensitivity of this subpopulation to metformin, as compared to “differentiated” tumor cells, determining the development of a drug repositioning approach (124, 125). In particular, while it is well-accepted that stem-like cells are particularly resistant to classical cytotoxic drugs due to the overexpression of drug-extruding pumps and DNA-repairing enzymes (110), in CSC cultures from different tumors the sensitivity to molecular-targeted drugs, in particular to tyrosine kinase inhibitors, is retained (114, 126).

In line with these premises, PASCs, isolated as SP, showed 1.5-fold up-regulation of the multidrug transporters ABCB1 and ABCG2 (73), thus suggesting their ability to extrude cytotoxic drugs. Accordingly, Xu et al. reported that PASCs from a GHoma were insensitive to both carboplatin and etoposide (69) and temozolomide resistance was described in another study (66). Interestingly, in a drug repositioning study, *in vitro* and *in vivo* treatment with disulfiram, a clinically approved drug for the treatment of alcoholism, sensitizes CD133^+^/nestin^+^ PASCs to temozolomide cytotoxicity, preventing drug-induced DNA damage repair by inhibiting O-6-methylguanine-DNA methyltransferase expression (83). Although CSC resistance to cytotoxic drugs is still a relevant issue in the treatment of all types of tumor, including aggressive PAs, PASCs might retain sensitivity to biological treatments. Thus, the sensitivity to commonly used drugs for PAs, somatostatin and dopamine agonists, and the expression of their receptors were tested in PASC subpopulations.

Both Wurth et al. and Peverelli et al. studies compared, in adenoma tissues and in PASC-enriched spheroids, the expression of somatostatin (SSTR) and dopamine (D2R, dopamine receptor type 2) receptors, which are targeted by currently used pharmacological treatments (i.e., SSTR2 and SSTR5, which are activated by lanreotide and octreotide, and D2R, activated by cabergoline) (77, 78). In the first study both SSTR2 and SSTR5 were expressed by the original GH-secreting tumors and within the analyzed sections, small groups of putative PASCs (immunopositive for NESTIN) also express SSTR2. Hence, it was suggested that these cells are not completely undifferentiated but could represent a progenitor cell subpopulation, expressing relevant regulatory neuropeptide receptors. This possibility was confirmed by *in vitro* studies on PASC spheroids which were mainly formed by cells co-expressing NESTIN, SSTR2 and SSTR5; moreover, also OCT4^+^/D2R^+^ double positive cells were detected in spheroids (77). Similar results were obtained by Peverelli et al., who reported the expression of mRNAs for *SSTR2* and *D2R*, in both histological preparations and spheroids from NFPAs, evaluated by RT-PCR. Unexpectedly, *SSTR5* mRNA was undetectable in spheroids although expressed in all tissues analyzed (78). The expression of all the five *SSTRs* was analyzed and compared with the original tumors also in PA MSCs derived from six GHomas and six NFPAs (75), although a different picture was reported. Mesenchymal PASCs derived from both PA subtypes express *SSTR1* at the highest level, while GHomas show higher expression of *SSTR1–4–5* than NFPAs, and lower level of for *SSTR2–3* was detected in both subtypes.

PASC expression of these pharmacologically relevant receptors prompted the analysis of their possible modulation to induce antiproliferative effects. In NFPAs, the role of SSTR2 and D2R activation in mediating antiproliferative signals was analyzed using two selective agonists: BIM23120 for SSTR2 and BIM53097 for D2R (78). The analysis was performed by BrdU incorporation assay either soon after cell dispersion (day 3) or after 30 days *in vitro*, when PASC-containing spheres were completely formed. Interestingly, a different responsivity among the cultures was observed, with about 33% of tested adenomas showing reduced proliferation in response to BIM23120, and 43% to BIM53097. Sensitive tumors showed a similar response in both culture conditions, indicating that drug sensitivity was already present within tumors and not acquired during the *in vitro* selection and expansion. On the average, although variability among the cultures was observed, as expected, BIM23120 (10 nM for 72 h) reduced DNA synthesis in the spheroids by 65%, while BIM53097 by 45% (78). Moreover, both drugs increased spheroid cell expression of the cyclin-dependent kinase inhibitor p27^Kip1^ and decreased cyclin D3 content, as molecular correlate of the antiproliferative effects of these receptors, as already reported for SSTRs (127). Importantly, no difference in the frequency of sphere formation among drug sensitive and resistant adenomas was observed, although the former were larger in size (78). Wurth et al., in light of the co-expression of SSTR2, SSTR5, and D2R in the same spheroids, used the MTT reduction assay to test the antiproliferative effects mediated by these receptors using an innovative chimeric SSTR2/SSTR5/D2R agonist, named BIM23A760 (128). In all the cultures analyzed, BIM-23A760 (1 nM, for 24 h) inhibited growth of spheroid-derived PASCs, reaching a statistical significance in 6 out of 7 PAs (ranging from −14 to −30% of vehicle-treated controls), independently from the length of the period of growth *in vitro* (analogous results were obtained in cultures lasting from 7 to 30 days) (77). An indirect evidence of the antiproliferative activity of D2R in PASCs was provided in *D2R* knockout mice, which develop prolactinomas starting at 6–8 months of age, and whose tumors show higher colony forming activity and a 2.4-fold increase in Sox2^+^ cells than the WT glands (73). An unexpected difference in SSTR agonist sensitivity between mesenchymal PASCs derived from GHomas and NFPAs, was also reported (75), using native somatostatin (1 μM), which activates all the five SSTRs, octreotide, able to bind SSTR2 and SSTR5, and pasireotide, which activate all SSTRs but SSTR4. In fact, using both XTT test and direct cell count, a statistically significant inhibition of proliferation was observed with all the agonists in PASCs derived from GHomas after 72 or 144 h of treatment (max inhibition about−40% for both octreotide and pasireotide), without affecting EMT or the induction of apoptosis. Conversely, no effects were detected in mesenchymal PASC from NFPAs, although SSTRs were expressed in these cells, and previous studies reported antiproliferative activity of somatostatin, octreotide, and pasireotide (112, 129, 130).

Finally, a different experimental model using PASCs isolated as SP from tumors originating from the murine ACTHoma cell line AtT20 (73) was used to demonstrate the role of the chemokine CXCL12 (131) in PASC proliferation. The inhibition of CXCR4, the CXCL12 receptor, using the clinical approved CXCR4 antagonist plerixafor, reduced EMT-associated PASC motility *in vitro*, and xenograft tumor growth *in vivo* (73).

All these data (summarized in Table 2) support the notion that in PASCs, although isolated from different PA subtypes, with different procedures, showing an undifferentiated phenotype, express most of the membrane receptor systems involved in pituitary cell stimulation (CXCR4) or inhibition (SSTRs, D2R) of cell proliferation are expressed and functioning. Thus, their modulation may represent a valuable pharmacological goal for this otherwise drug-resistant subpopulation.

**Table 2:** Drug sensitivity of pituitary adenoma stem cells.

**PASC origin**	**PA histotype**	**Drug**	**Target (s)**	**Effect**	**References**
HUMAN	GH-oma	Carboplatin	DNA alkylation	Drug resistance	(69)
		Etoposide	Topoisomerase II	Drug resistance	
HUMAN	GH-oma	Temozolomide	DNA alkylation	Drug resistance	(66)
HUMAN	n.s.	Temozolomide	DNA alkylation	Drug resistance (MGMT-dependent)	(83)
		Disulfiram	ALDH	Cell sensitization to temozolomide	
HUMAN	NFPA	BIM23120	SSTR2	Anti-proliferative	(78)
		BIM53097	D2R	Anti-proliferative	
HUMAN	NFPA	BIM23A760	SSTR2,5-D2R	Anti-proliferative	(77)
HUMAN	GH-oma	Somatostatin	SSTR1-5	Anti-proliferative	(75)
		Octreotide	SSTR2,5	Anti-proliferative	
		Pasireotide	SSTR1,2,3,5	Anti-proliferative	
HUMAN	NFPA	Somatostatin	SSTR1-5	No effect	(75)
		Octreotide	SSTR2,5	No effect	
		Pasireotide	SSTR1,2,3,5	No effect	
MOUSE	AtT20 cells (ACTH-oma)	Plerixafor	CXCR4	Inhibition of EMT-associated motility and xenograft tumor growth	(73)

*PASC, pituitary adenoma stem cell; PA, pituitary adenoma; MGMT, O-6-methylguanine-DNA methyltransferase; ALDH, aldehyde dehydrogenase; SSTR, somatostatin receptor; D2R, dopamine receptor 2; n.s., not specified*.

## Conclusions and Future Perspectives

From the literature we reviewed it is clear that, although consistent information is currently available concerning the actual existence of PASCs, many unsolved questions will need to be further explored.

Notwithstanding the highlighted differences among the published studies, the presence of stem-like cells in benign tumors seems to be a confirmed notion. In particular, the reported cell heterogeneity in PAs, a tumor type which by definition is considered monoclonal in origin, further reinforces this assumption. However, the divergent differentiation fate of PA cells during tumor development (retaining of stem-like phenotype, differentiation in hormone producing cells, acquisition of mesenchymal or neural features) rises the relevant issue of which factors actually control this transition, and the identification of possible stem cell niche(s) within the tumor mass where PASC can self-renew and act as reservoir for the bulk of tumor cells. To date, although some tumor areas were identified as putative PA niches, no formal demonstration has been provided. Importantly, although discrepancies among the papers that analyzed PASC features are clearly related to the procedures (different permissive media, side-populations, etc.) or the model (human PA of different subtypes, or mouse tumors) used, it is also evident that multiple stem-like cell populations may exist within PAs. Thus, it is very important to define the characteristics of these cells not only as far as the biological features are concerned, but also to address whether different PASC subpopulations co-exist in the same tumor or different PAs develop from stem-like cells with distinct phenotypes. In this respect, another unsolved issue is the actual cell of origin (the real tumor stem cell) of PAs. While in the past most evidence was pointing out the role of FS cells, now it seems more likely that oncogenic transformation should occur in adult PSCs, or more differentiated pituitary progenitors. In this respect, novel technologies, including single cell RNA sequencing, may provide a significant contribution to address the question regarding the origin of different PASC populations within PAs, as recently performed in adult pituitaries (132). In this context, the complete characterization of the different PA subpopulations will allow the identification of the bases of the differential efficacy of cytotoxic and biological pharmacological treatments on PASCs. In fact, although cytotoxic drugs are scarcely effective on PASCs, as expected in putative CSCs, these cells express SSTRs and D2R and are responsive to the respective agonists. Considering that the clinically approved receptor agonists, such as octreotide, lanreotide, pasireotide, and cabergoline, display both anti-secretory and, although less defined, antiproliferative activities (133), it is conceivable that these dual effects may involve the modulation of receptors in different PA cell subsets: the former likely mainly acting on differentiated cells, and the latter on PASCs. Further studies trying to clarify this issue are most warranted.

Another relevant concern is the identification of mechanisms controlling PASC proliferation, self-renewal, and differentiation. Recently a role of miRNA in pituitary tumorigenesis was proposed (134, 135). miRNAs are small non-coding RNAs that post-transcriptionally modulate gene expression, and whose expression is altered in several human neoplasia. For example, relevant for the topic of this review, a different miRNA expression pattern was observed in NFPAs and normal pituitary (136) with a significant influence on stem cell-related pathways, such as NOTCH (137). The field of miRNA is rapidly evolving and its application to different PASC populations might provide important novel information concerning the pathogenic mechanisms activated in these cells to originate PAs.

Finally, a crucial issue for PASC research is the demonstration of their tumorigenic potential, a required feature to define putative CSCs. The development of animal models using human PA cells is also extremely relevant to provide pharmacological preclinical clues to be translated in clinical studies. As discussed in the previous paragraph, this topic is very puzzling using traditional mouse models, and novel approaches, such as zebrafish embryo, can address only few aspects of this matter (i.e., neoangiogenesis and cell migration). Recently, development of 3D organoid systems (63), derived from Sox2-expressing normal mouse pituitary cells allowed to maintain long-term growth and stem-like phenotype during the expansive culture but also to the ability to differentiate into hormone-producing subpopulations. The application of this methodology to PAs may contribute to definition of the actual tumor-initiating subpopulation, the cellular and molecular pathways underlying tumor growth and the possible acquisition of invasive features, overcoming limitations observed using animal models.

## Author Contributions

All authors contributed to the literature search and selection, the critical analysis of the data, and to writing the manuscript.

### Conflict of Interest

The authors declare that the research was conducted in the absence of any commercial or financial relationships that could be construed as a potential conflict of interest.

## Abbreviations

HES: Hairy Enhancer of Split
HEY: HES-related with YRPW motif
Smo: Smoothened
Ptch1: Patched
GLI: Gli zinc finger transcription factors
LATS1/2: large tumor suppressor homolog 1 and 2
YAP: Yes-associated protein
TAZ: Transcriptional co-activator with PDZ binding motif
TEAD: Transcriptional enhanced associate domain
FZD: Frizzled
LEF: Lymphoid enhancer factor
LGR: Leucine rich repeat containing G protein-coupled receptor
TCF7L1: Transcription factor 7 like 1
CXCR4/7: CX-chemokine receptors 4 and 7
TGFRB2: Transforming Growth Factor Beta-Receptor 2
TWIST1: Twist-related protein 1
ZEB1: Zinc-finger E-box-binding homeobox 1.
